# Micropatterned Poly(3,4-ethylenedioxythiophene) Thin Films with Improved Color-Switching Rates and Coloration Efficiency

**DOI:** 10.3390/polym14142951

**Published:** 2022-07-21

**Authors:** Cheng-Lan Lin, Tzung-Lin Cheng, Nian-Jheng Wu

**Affiliations:** 1 Department of Chemical and Materials Engineering, Tamkang University, New Taipei City 25137, Taiwan; 135800@mail.tku.edu.tw; 2 Water Treatment Science and Technology Research Center, Tamkang University, New Taipei City 25137, Taiwan; 3 CNRS, Institut des Sciences Moléculaires d’Orsay, Université Paris-Saclay, 91405 Orsay, France; nianjheng.wu@u-psud.fr

**Keywords:** poly(3,4-ethylenedioxythiophene), micropattern, electrochromic material, electrode

## Abstract

Electrochromic materials carry out redox reactions and change their colors upon external bias. These materials are the primary component in constructing smart windows for energy saving in buildings or vehicles. Enhancing the electrochromic performances of the materials is crucial for their practical applications. Micropatterned poly(3,4-ethylenedioxythiophene) (mPEDOT) thin films are electrodeposited on indium tin oxide conducting glass in this study. Their electrochromic properties, including transmittance modulation ability, color-switching rates, and coloration efficiency, are investigated and compared with nonpatterned PEDOT thin films. The mPEDOT thin films exhibited faster coloring and bleaching speeds and higher coloration efficiency than the PEDOT thin films while keeping similar transmittance modulation ability. The results suggest that micropatterning an electrochromic material thin film might enhance its electrochromic performances. This research demonstrates the possibility of promoting the color-switching rate of a PEDOT thin film by micropatterning it.

## 1. Introduction

Electrochromic (EC) materials are species that can switch their absorptivity toward incident light upon external bias switches [1]. EC materials can be prepared as thin films on transparent conducting substrates, and the resulting electrodes can be used to construct electrochromic devices (ECDs). The operating mechanisms of ECDs are used for constructing smart window systems [2]. The systems manipulate light and heat interchange between a building or a vehicle and its surroundings. For example, reducing the heat transfer into a building through windows during summertime could decrease the loading of the air-conditioning system, which could achieve energy-saving purposes [3]. One of the major challenges in developing ECDs for practical applications is to improve the color-switching rate. Decreasing the coloring and bleaching response times of EC material thin films plays a pivotal role in promoting ECD performances. 

Poly(3,4-ethylenedioxythiophene) (PEDOT) is a well-known conducting polymer [4]. PEDOT derivatives electrodeposited from various functionalized 3,4-ethylenedioxythiophenes exhibit the possibility for multiple and a wide variety of color changes [5,6,7]. Their applications in many areas, such as energy conversion and storage devices [8], sensors [9], and displays [10], have been extensively explored. PEDOT is a polymeric EC material that can be switched between light-blue and dark-blue states. The coloring-switching mechanism corresponds to its electrochemical redox reactions [11]. The redox reactions of the PEDOT thin film can be expressed as Equation (1).

(1) $${\mathrm{PEDOT}}^{n+}{:\mathrm{nClO}}_{4\;}^{-}{+\mathrm{ne}}^{-}\;⇄{\;\mathrm{PEDOT}+\mathrm{nClO}}_{4}^{-}$$

The perchlorate ions in the electrolyte serve as the dopant during the reduction of the PEDOT. Electrons and perchlorate ions are inserted into the PEDOT thin film simultaneously to maintain the thin film’s natural state and are extracted from the thin film during oxidation. Efforts have been attempted to improve the coloring-switching rate of PEDOT electrodes, such as structure modifications [12,13,14] or incorporating other species [15,16,17]. Micropatterned PEDOT electrodes have been sporadically studied for sensor applications or optical properties [18,19]. Various methods have been employed for the fabrication of conjugated polymers, such as electropolymerization [20], oxidative chemical vapor deposition [21], and vapor phase polymerization [22]. Our previous studies developed the direct electrodeposition of micropatterned thin films [23,24]. This study prepared micropatterned PEDOT (mPEDOT) thin films on indium tin oxide (ITO) conducting glass by the direct micropattern electrodeposition method. The primary purpose was to investigate the effects of a micrometer-scale PEDOT thin film structure modification on its electrochromic properties. The bleached state of a PEDOT thin film is light blue. A micropatterned PEDOT thin film is also expected to provide higher bleaching state see-through visibility. Electrochromic properties of the mPEDOT thin films were investigated and compared with non-micropatterned PEDOT thin films prepared using the same procedures.

## 2. Materials and Methods

3,4-Ethylenedioxythiophene (EDOT) was purchased from Tokyo Chemical Industry. Acetonitrile (ACN) was obtained from Acros. Lithium perchlorate (LiClO_4_) and propylene carbonate (PC) was acquired from Alfa Aesar. Perchloric acid (HClO_4_) was bought from Sigma-Aldrich. All chemicals were used as received without further purification. A micropatterned electrostatic film (PE-1101E) was purchased from Youlen Technology Co., Ltd., New Taipei City, Taiwan) ITO (sheet resistance = 7 Ω/sq) conducting glass was obtained from AimCore Technology Co., Ltd., Hsinchu County, Taiwan.

**Figure 1:**
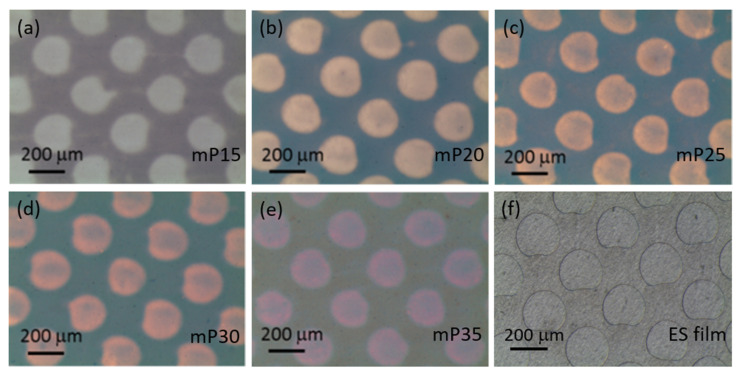
Optical microscope images of the mPEDOT thin films deposited using charge capacities of (**a**) 15 mC/cm^2^, (**b**) 20 mC/cm^2^, (**c**) 25 mC/cm^2^, (**d**) 30 mC/cm^2^, and (**e**) 35 mC/cm^2^. (**f**) Optical microscope image of an electrostatic film.

**Scheme 1:**
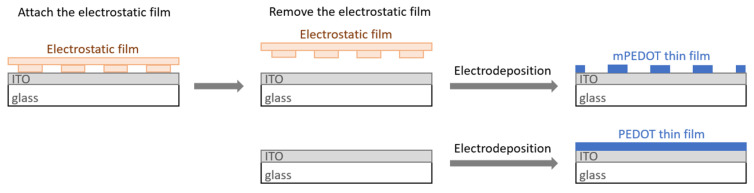
Illustration of the preparation procedures of the mPEDOT and PEDOT thin films.

For the electrodeposition of mPEDOT thin films, ITO conducting was successively washed with a neutral detergent solution, deionized water (DIW), and acetone in an ultrasonic bath each for 5 min. A micropatterned electrostatic (ES) film was attached to the ITO surface for more than 7 days. The ES film consisted of an array of polyacrylate circle bumps (diameter ~ 220 μm) on a polyethylene sheet. Figure 1f shows an optical microscope image of the micropatterned ES film. The preparation procedures of the mPEDOT and PEDOT thin films are illustrated in Scheme 1. The ES film was removed before the electrodeposition of PEDOT. Polyimide tapes fixed the ITO surface area to be 1.0 × 1.0 cm^2^. The currents measured in the electrochemical experiments can be expressed as current density since the working area was 1.0 cm^2^. The plating solution for the electrodeposition of PEDOT thin films was an ACN solution containing 0.01 M EDOT and 0.1 M LiClO_4_. A constant potential of 1.2 V (vs. Ag/Ag^+^) was applied until the passing charge capacity was 15, 20, 25, 30, or 35 mC/cm^2^. Bluish mPEDOT thin films were then obtained and named mP15, mP20, mP25, mP30, and mP35. Before further experiments, the thin films were washed by ACN, dried, and stored in an electronic dry cabinet. Non-micropatterned PEDOT thin films were also prepared on a pristine ITO substrate using the same electrodeposition procedures for comparisons. These thin films were P15, P20, P25, P30, and P35.

The thickness of the mPEDOT and PEDOT thin films was measured by a surfcorder (EZSTEP, Force Precision Instrument Co., Ltd., New Taipei City, Taiwan). A blade was used to scratch off the mPEDOT or PEDOT thin film and reveal the ITO surface for the film thickness measurements. The surfcorder tip was started from the ITO surface and moved toward the mPEDOT or the PEDOT thin film, and the height variations were recorded. The tip moving path is shown in Scheme S1 (Supporting Information). An optical microscope (BX51, Yuan Li Instrument Co., Ltd., Taipei City, Taiwan) was used to obtain the surface images of the thin films. A conventional three-electrode system with a potentiostat/galvanostat (CHI 611D, CH Instruments, Inc., Austin, TX, USA) was employed for the electrochemical experiments. The reference electrode was an Ag/Ag^+^ electrode, and the counter electrode was a platinum foil. Cyclic voltammetry and potential-step experiments were carried out in a PC solution containing 0.1 M LiClO_4_ and 1.0 mM HClO_4_. Transmittance spectra of the thin films were acquired by a UV–VIS spectrometer (SD1200-LA-HA, OTO Photonics Instruments Co., Hsinchu City, Taiwan). In situ experiments obtained transmittance changes during potential-step operations of the thin films. The UV–VIS spectrometer recorded the transmittance changes, while the potentiostat applied different potentials to the thin films. The obtained data were used to estimate the color-switching response times of the thin films.

## 3. Results and Discussion

mPEDOTs are prepared on electrostatic film-pretreated ITO substrates with different electrodeposition charge capacities (Q_d_). The PEDOT thin film was mainly deposited on the ITO surface, which was not attached by the polyacrylate bumps of the ES film (outside the light-colored circles in Figure 1a–e). Their optical microscope images are shown in Figure 1. The deep-colored (bluish) portions indicate the distribution of the deposited PEDOT thin films. The light-colored parts correspond to the areas with comparatively fewer PEDOTs deposited. The light-colored circles were arranged regularly as an array. The circle diameter was about 200 μm, and the distance between the circle centers was about 300 μm. The size of the circles and their arrangements consisted of the bump distribution of the electrostatic film. It can be observed that a small amount of PEDOT started to be deposited within the light-colored circle areas as the deposition charge capacities (Q_d_) increased from 15 to 35 mC/cm^2^. The optical microscope images indicated that micropatterned PEDOT thin films could be successfully deposited on the ITO surface. The dense PEDOT thin film covered about 60% of the ITO substrate surface.

**Figure 2:**
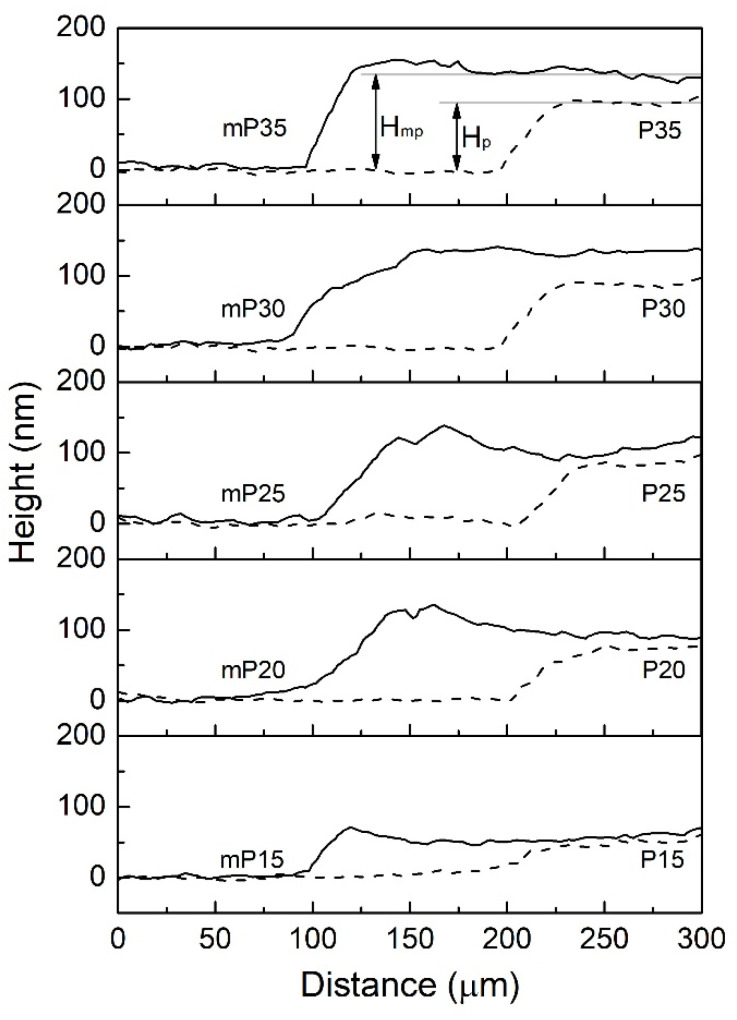
Thickness of the mPEDOT and PEDOT thin films.

A surface profiler measured the thickness of the PEDOT and mPEDOT thin films prepared using different Q_d_’s. The results are shown in Figure 2 and listed in Table 1. The average thickness of the mPEDOT thin films at the deep-colored area (H_mP_) was higher than the average thickness of the PEDOT thin films (H_P_) obtained using the same Q_d_. The PEDOT thickness within the light-colored circle was negligible compared with H_mP_. According to the OM images and the measured film thickness, the PEDOT deposition was inhibited on the ITO surface areas attached by the bumps of the electrostatic film. The electropolymerization of PEDOT concentrated on the un-pretreated ITO surface areas. 

**Figure 3:**
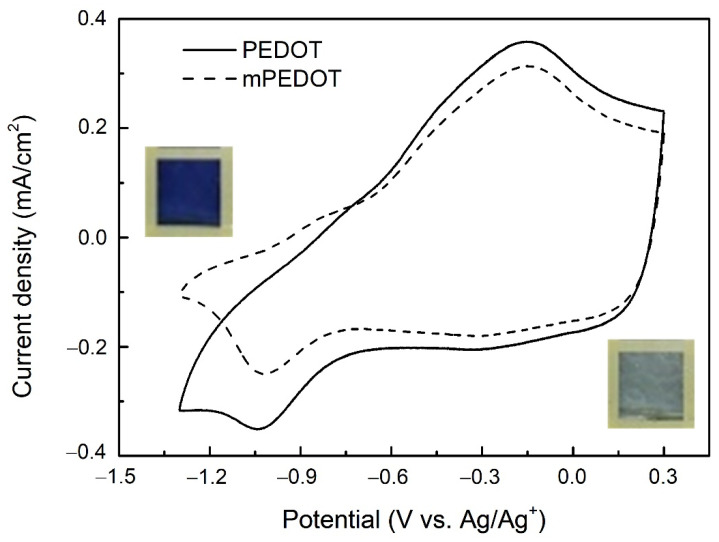
Cyclic voltammograms of the mPEDOT and PEDOT thin films prepared using Q_d_ of 20 mC/cm^2^. The scan rate was 100 mV/s. The electrolyte was a PC solution containing 0.1 M LiClO_4_ and 1.0 mM HClO_4_. The inset optical images are the colored state (left side) and the bleached state (right side) images of the mPEDOT thin film.

Figure 3 shows the typical cyclic voltammograms of the mPEDOT and PEDOT thin films (Q_d_ = 20 mC/cm^2^). The current responses increased with the increasing Q_d_ used in the electrodeposition process for both thin films. During the cathodic potential scan, the thin films changed from light-blue to deep-blue color from 0.3 V (vs. Ag/Ag^+^) to −1.3 V (vs. Ag/Ag^+^). The thin-film bleaching process took place during the reverse potential scan. Current responses of the mPEDOT thin films were smaller than those of the PEDOT thin films prepared using the same Q_d_. Smaller current responses may imply that a fewer PEDOTs were polymerized on the pretreated ITO substrate. 

The coloration charge capacities of the mPEDOT and PEDOT thin films (Q_c,mP_ and Q_c,P_) were estimated by potential-step experiments. Successive chronoamperometry experiments were carried out to estimate Q_c,mP,_ and Q_c,P_. The resulting *i*-*t* curves are shown in Figures S1 and S2 (Supporting Information). The thin films were switched from their bleached state (0.3 V (vs. Ag/Ag^+^)) to their colored state (−1.3 V (vs. Ag/Ag^+^)), and the corresponding Q_c,mP_ and Q_c,P_ were obtained from the integration of the *i*-*t* curves. The results are listed in Table 2. Both Q_c,mP_ and Q_c,P_ increased with Q_d_, and Q_c,mP_ was larger than Q_c,P_. The relative percentage difference (RPD) of the coloration charge capacity, which is defined as ((Q_c,mP_ − Q_c,P_)/Q_c,P_) × 100%, was calculated and is listed in Table 2. RPD became smaller as larger Q_d_ was used for the electrodeposition process. The observation indicated that fewer PEDOTs were formed on electrostatic film-pretreated ITO surfaces. Additionally, the influence of the pretreatment ITO surface on the electrodeposition process diminished as the thicker film was prepared (more extensive Q_d_ employed).

The surface coverage of the mPEDOT thin films was about 40% smaller than that of the PEDOT thin films, but their coloration charge capacity differences were smaller than 20%. With the same Q_d_, the obtained mPEDOT thin films were thicker than the PEDOT thin films, as shown in Table 1. These results suggest that the electropolymerization process was concentrated on the ITO surfaces attached by the electrostatic film. The PEDOT electrodeposition was enhanced on the electrostatic film attached to ITO surface areas. The uneven surface electropolymerization processes thus formed the micropatterned PEDOT thin films.

**Figure 4:**
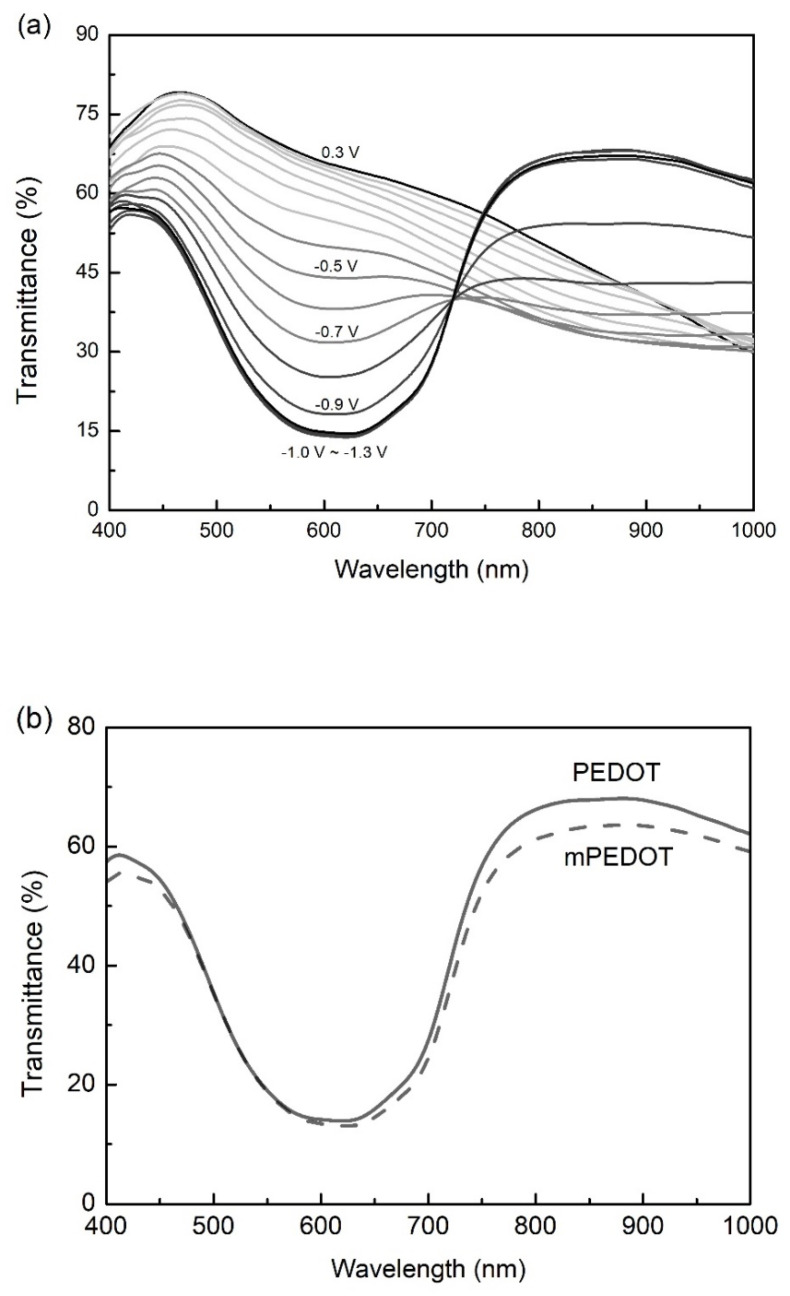

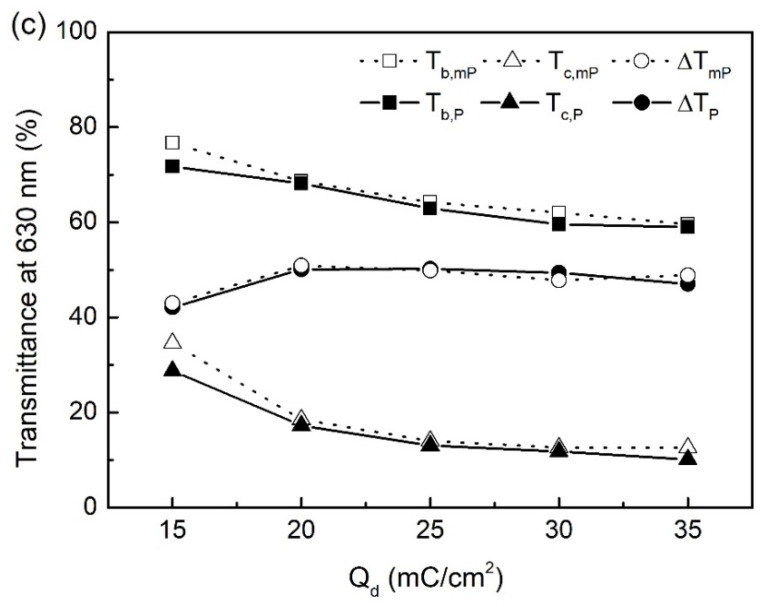
(**a**) Typical transmittance spectra of the PEDOT thin film (Q_d_ = 25 mC/cm^2^) at different applying potentials. (**b**) Comparisons of the colored-state transmittance spectra of the mPEDOT and PEDOT thin films. (**c**) Comparisons of T_c_, T_b_, and ΔT (at 630 nm) of the mPEDOT and PEDOT thin films. The coloring and bleaching potentials were −1.1 V (vs. Ag/Ag^+^) and 0.3 V (vs. Ag/Ag^+^), respectively. The electrolyte was a PC solution containing 0.1 M LiClO_4_ and 1.0 mM HClO_4_.

Figure 4a shows the typical transmittance spectra of the PEDOT thin film at different applying potentials. The applying potential difference between each spectrum was 0.1 V. The PEDOT thin film was at its bleaching state when the applying potential was 0.3 V (vs. Ag/Ag^+^). Minor transmittance changes were observed when the applying potential varied from 0.3 to 0.0 V (vs. Ag/Ag^+^). The apparent overall thin film transmittance decrease started as the applying potential switched from 0.0 to −1.0 V (vs. Ag/Ag^+^). The PEDOT thin film was at its fully colored state when the applying potential was more negative than −1.0 V. As can be seen in Figure 4, negligible spectra differences were recorded for the applying potential between −1.0 V (vs. Ag/Ag^+^) and −1.3 V (vs. Ag/Ag^+^). The mPEDOT thin films had similar transmittance spectra as the PEDOT thin films since they were electrodeposited using the same procedures. 

Representative colored-state spectra of the mPEDOT and PEDOT thin films at an applying potential of −1.1 V (vs. Ag/Ag^+^) are compared in Figure 4b. The overall colored-state transmittance decreased as larger Q_d_ was used in the electrodeposition process. The mPEDOT thin films showed a slightly higher colored-state transmittance than the PEDOT thin films prepared using the same Q_d_. Differences between the colored-state and bleached-state transmittance of the mPEDOT thin films (T_c,mP_ and T_b,mP_) and PEDOT thin films (T_c,P_ and T_b,P_) at 630 nm are compared in Figure 4c. Both coloring and bleaching response times decreased with the increasing Q_d_. The transmittance modulation abilities of the mPEDOT and PEDOT thin films, ΔT_mP_ and ΔT_P_, were defined as the transmittance difference between their colored and bleached states (T_b,mP_ − T_c,mP_ or T_b,P_ − T_c,P_). Table 3 lists the measured colored and bleached state transmittances and corresponding transmittance modulation abilities at 630 nm. The mPEDOT thin film has a higher colored-state and bleached-state transmittance than the PEDOT thin films. The mPEDOT thin film covered about 60% of the ITO surface, allowing more incident light to pass through it. Therefore, the mPEDOT thin films could have higher bleached and colored state transmittance than a non-micropatterned PEDOT thin film. Similar ΔT_mp_ and ΔT_p_ were obtained when the mPEDOT and PEDOT thin films were electrodeposited using the same Q_d_. On average, 50% of ΔT_mp_ or ΔT_p_ can be achieved by these thin films (except those prepared using Q_d_ = 15 mC/cm^2^). The mPEDOT thin films have lower ITO surface coverage than the PEDOT thin films. It is deduced that their larger film thicknesses (as shown in Figure 2 and Table 1) might contribute to more local transmittance changes. Therefore, these thin films achieved similar overall transmittance modulation ability. 

**Figure 5:**
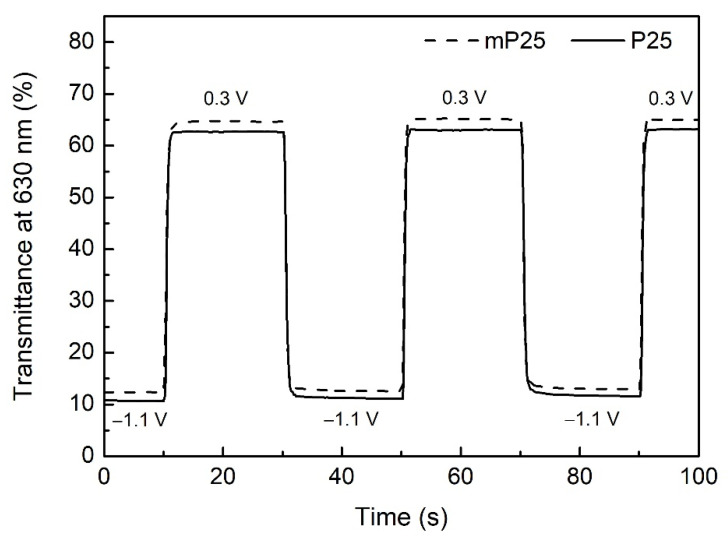
Transmittance responses (at 630 nm) of the mP25 and the P25 thin films during successive potential switches between their colored state (−1.1 V vs. Ag/Ag^+^) and bleached state (0.3 V vs. Ag/Ag^+^). The electrolyte was a PC solution containing 0.1 M LiClO_4_ and 1.0 mM HClO_4_.

The thin films’ coloring or bleaching response times (t_c_ or t_b_) were defined as the times needed to achieve 90% of their fully colored or bleached state. Successive potential switches were carried out between the colored and bleached states of the mPEDOT and PEDOT thin films. Typical in situ transmittance changes of the mP25 and P25 thin films (at 630 nm) during the potential switches are shown in Figure 5. The color-switching response times of the mPEDOT thin films (t_c__,mp_ and t_b__,mp_) and the PEDOT thin films (t_c__,p_ and t_b__,p_) were calculated, and the results are listed in Table 4. Both coloring and bleaching response times were prolonged with larger Q_d_, indicating that the increasing film thickness slowed the color-switching rates. The color-changing reaction involves the transportations of both electrons and ions between the electrolyte and the PEDOT thin film. It is deduced that ions need more time to migrate within a thicker PEDOT film and decreased color-switching rate. 

**Figure 6:**
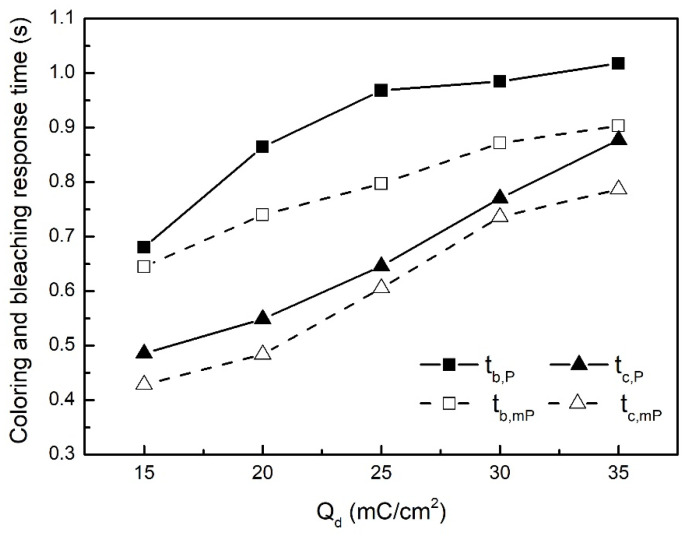
Comparison of the coloring and bleaching response times of the mPEDOT and PEDOT thin films prepared using different Q_d_’s.

Figure 6 compares the coloring and bleaching response times of the mPEDOT and PEDOT thin films prepared using the same Q_d_. The thin films’ bleaching response times (tb,mP and tb,P) were shorter than their coloring response times (t_c,mP_ and t_c,P_). The mPEDOT thin films had faster coloring switching speeds than the PEDOT thin films. According to these results, it is deduced that micropatterning a PEDOT thin film might slightly facilitate its color-switching rates. 

**Figure 7:**
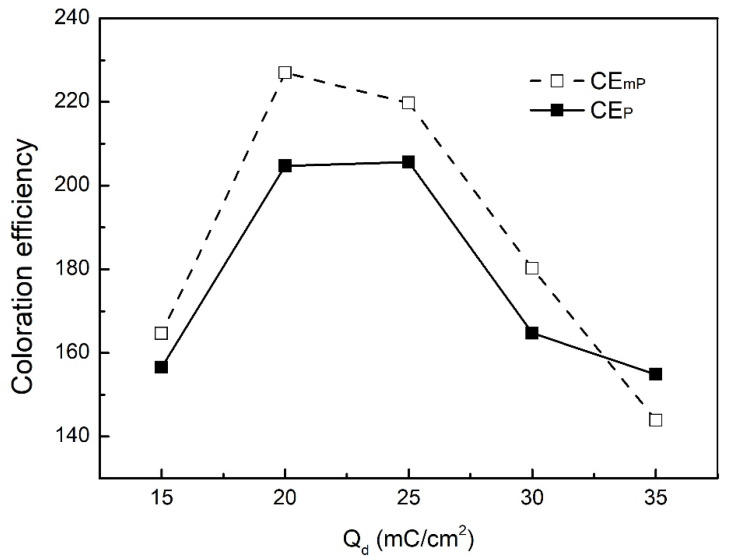
Comparison of the coloration efficiency of the mPEDOT and PEDOT thin films prepared using different Q_d_’s.

An electrochromic thin film’s color efficiency (CE) is defined as ΔOD/Q_c_ [25]. ΔOD is the optical density estimated from the difference in the absorbance of the colored and bleached state of an electrochromic thin film. The CE of the mPEDOT and PEDOT thin films were calculated, and the results are shown in Figure 7. A larger CE value is desired for an electrochromic thin film since unit energy consumption can result in a more significant absorbance change. It is noted that the mPEDOT thin films have higher CE than the PEDOT thin films (except for the thin film prepared using Q_d_ = 35 mC/cm^2^). For the thin films prepared using the same Q_d_, the mPEDOT thin films have minor Q_c_ (Table 2) but similar ΔT (Table 3) compared with the PEDOT thin films. It is deduced that micropatterning of a PEDOT thin film might facilitate its redox reactions while keeping its transmittance modulation ability, resulting in higher CE and more energy-saving possibilities. 

## 4. Conclusions

Micropatterned PEDOT thin films were directly electrodeposited on ITO substrates using different Q_d_’s. The electrochromic properties of these thin films were investigated and compared with those of the PEDOT thin films prepared using the same Q_d_. These thin films have similar ΔT, but the mPEDOT thin film has higher colored-state and bleached-state transmittance than the PEDOT thin films. The mPEDOT thin films have lower ITO surface coverage but larger film thickness, which should be the reason for having similar ΔT as the PEDOT thin films prepared using the same Q_d_. It is noted that both the coloring and bleaching response times of the mPEDOT thin films were faster than those of the corresponding PEDOT thin films. Additionally, the mPEDOT thin films have higher CE than the PEDOT thin films. These results suggest that micropatterning of a PEDOT thin film could preserve its transmittance modulation ability and, at the same time, increase its coloring-switching rates and CE [19,26]. It is envisaged that such a strategy could serve as a promising method to improve the performance of electrochromic thin film electrodes. Further studies on the performance influences of the electrochromic devices using these micropatterned electrodes were undergoing in our laboratory. 

## Figures and Tables

**Table 1:** Thickness of the mPEDOT and PEDOT thin films.

Q_d_ (mC/cm^2^)	mPEDOT H_mP_ (nm)	PEDOT H_P_ (nm)
15	55	50
20	96	73
25	109	82
30	132	89
35	137	96

**Table 2:** Coloration charge capacities of the mPEDOT and PEDOT thin films prepared using different Q_d_’s.

Q_d_ (mC/cm^2^)	Q_c,mP_ (mC/cm^2^)	Q_c,P_ (mC/cm^2^)	RPD (%)
15	2.10	2.54	−17.3%
20	2.51	2.91	−13.7%
25	3.01	3.33	−9.6%
30	3.84	4.27	−10.1%
35	4.69	4.95	−5.3%

**Table 3:** Colored-state and bleached-state transmittance and transmittance modulation ability of the mPEDOT and PEDOT thin films electrodeposited using different Q_d_.

Q_d_ (mC/cm^2^)	mPEDOT			PEDOT		
	T_b,mP_ (%)	T_c,mP_ (%)	ΔT_mP_ (%)	T_b,P_ (%)	T_c,P_ (%)	ΔT_P_ (%)
15	76.7	34.6	42.1	71.7	28.7	43.0
20	68.7	18.5	50.1	68.2	17.3	50.9
25	64.2	14.0	50.2	62.9	13.0	49.9
30	62.0	12.6	49.4	59.6	11.8	47.8
35	59.6	12.6	47.0	59.0	10.1	48.9

ΔT_mP_ = T_b,mP_ − T_c,mP_; ΔT_P_ = T_b,P_ − T_c,P_.

**Table 4:** Coloring and bleaching response times of the mPEDOT and PEDOT thin films electrodeposited with different Q_d_’s.

Q_d_ (mC/cm^2^)	mPEDOT		PEDOT	
	t_c,mP_ (s)	t_b,mP_ (s)	t_c,P_ (s)	t_b,P_ (s)
15	0.65	0.43	0.68	0.49
20	0.74	0.48	0.86	0.55
25	0.80	0.61	0.97	0.65
30	0.87	0.74	0.99	0.77
35	0.90	0.79	1.02	0.88

## Data Availability

The data presented in this study are available on request from the corresponding author.

## Supplementary Materials

The following supporting information can be downloaded at: https://www.mdpi.com/article/10.3390/polym14142951/s1, Scheme S1: Illustration of the surfcorder tip moving path when measuring the thickness of a mPEDOT thin film. Figure S1: *i-t* curves of the successive chronoamperometry experiments of the PEDOT thin films electrodeposited using different Q_d_.; Figure S2: *i-t* curves of the successive chronoamperometry experiments of the mPEDOT thin films electrodeposited using different Q_d_.
