# Supplementary material for: Micropatterned Poly(3,4-ethylenedioxythiophene) Thin Films with Improved Color-Switching Rates and Coloration Efficiency
Source: Polymers (Basel). 2022 Jul 21;14(14):2951. doi: 10.3390/polym14142951 (PMC9320408; doi:10.3390/polym14142951)
Supplement: Supplementary file 1 [file polymers-14-02951-s001.zip › polymers-1787379-supplementary.pdf]

Micropatterned Poly(3,4-ethylenedioxythiophene) Thin films with Improved Color-switching Rates and Coloration Efficiency

Cheng-Lan Lin[a,b,*], Tzung-Lin Cheng[a], Nian-Jheng Wu[c]

[a] Department of Chemical and Materials Engineering, Tamkang University, New Taipei City, Taiwan
[b] Water Treatment Science and Technology Research Center, Tamkang University, New Taipei City, Taiwan
[c] Institut des Sciences Moléculaires d'Orsay, Université Paris-Saclay, CNRS, Orsay, France

*Corresponding author. Department of Chemical and Materials Engineering, Tamkang University, New Taipei City 25137, Taiwan. Tel.: + 886 2 26215656 ext. 2723; E-mail address: cllin@mail.tku.edu.tw; cllintw@gmail.com.

Supporting Information

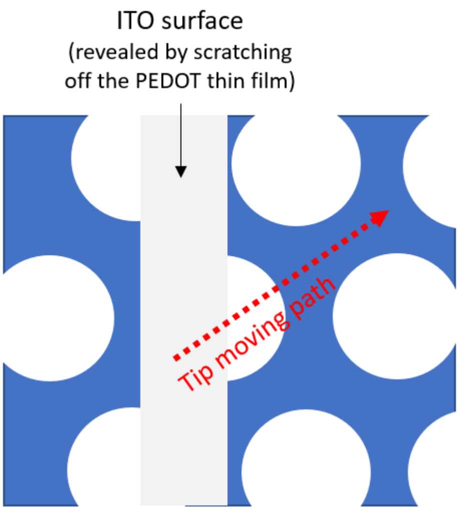

Scheme S1 Illustration of the surfcorder tip moving path when measuring the thickness of a mPEDOT thin film.

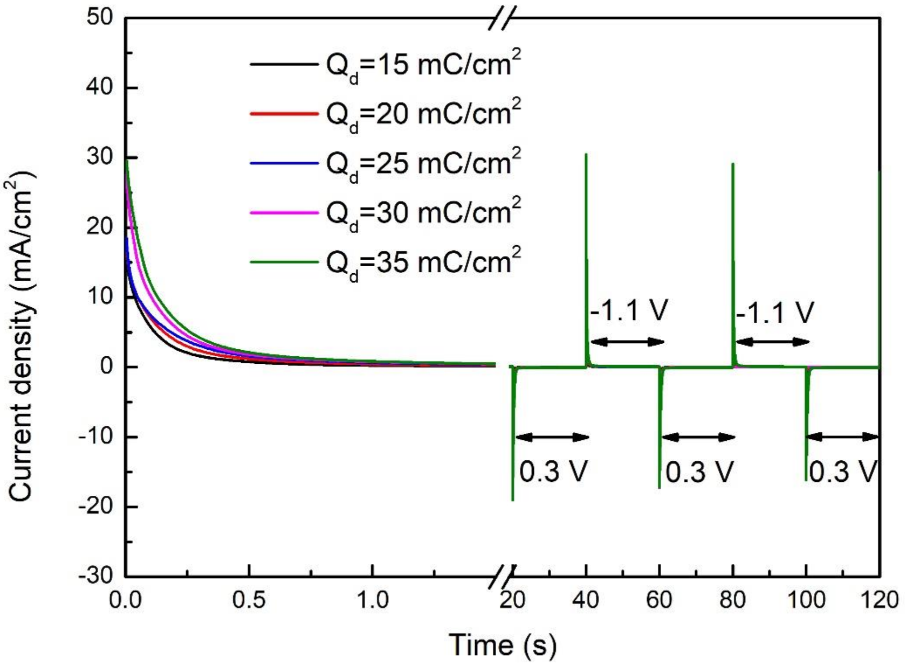

Figure S1 *i-t* curves of the successive chronoamperometry experiments of the PEDOT thin films electrodeposited using different Qd.

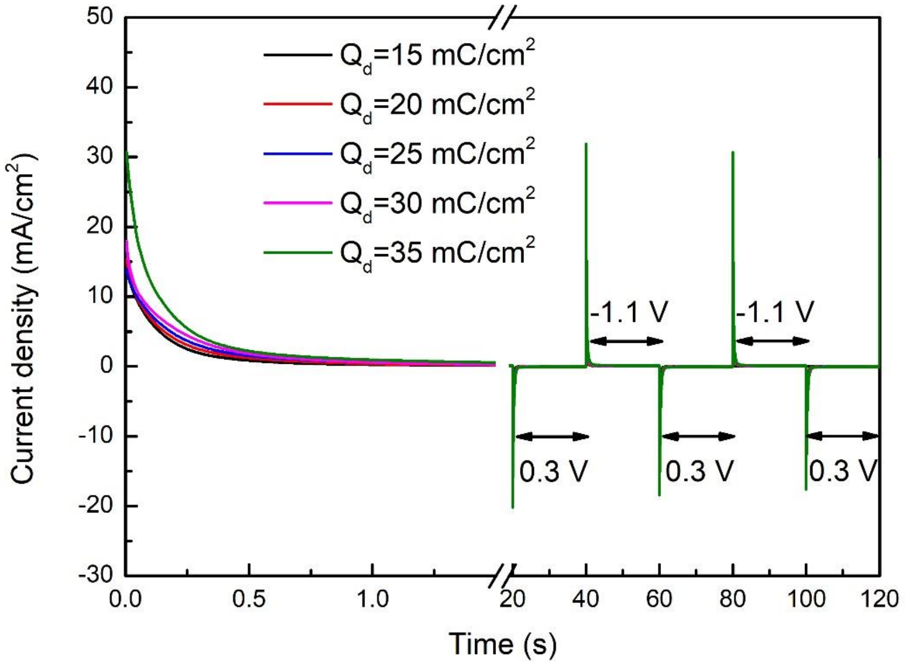

Figure S2 *i-t* curves of the successive chronoamperometry experiments of the mPEDOT thin films electrodeposited using different Qd.
